# Late-Onset Ileocutaneous Fistula Eight Years After Plug Repair With Polypropylene Mesh: A Case Report

**DOI:** 10.3389/fsurg.2021.785087

**Published:** 2021-11-15

**Authors:** Jiankang Zhang, Zeming Hu, Xuan Lin, Bin Chen

**Affiliations:** ^1^Department of General Surgery, The First Affiliated Hospital of Gannan Medical University, Ganzhou, China; ^2^Department of General Surgery, The First Affiliated Hospital of Nanjing Medical University, Nanjing, China

**Keywords:** laparoscopic exploration, mesh, late-onset, inguinal hernia, intestinal fistula, case report

## Abstract

**Introduction:** As one of the short-term complications after inguinal hernia repair, mesh infection frequently occurs but rarely leads to ileocutaneous fistula. We present a rare case of ileocutaneous fistula 8 years after inguinal hernia plug repair with polypropylene mesh.

**Case Presentation:** The patient was a 67-year-old male who underwent a plug repair with polypropylene mesh of the right inguinal hernia. Eight years after the primary repair, skin ulceration with pus presented in the right groin area, and the final diagnosis was enterocutaneous fistula. According to laparoscopic exploration, the ileum below the fistula closely adhered to the abdominal wall. After gently separating the bowel loop, a defect area of about 2 × 3 cm was observed on the surface of the ileum. In laparotomy, the plug was found embedded in the ileum and then was completely removed, and an ileum side-to-side anastomosis was performed. The patient was discharged 2 weeks after the surgery, and follow-up at the sixth month revealed complete healing of the wound and no evidence of hernia recurrence.

**Conclusion:** Late-onset ileocutaneous fistula should be considered in the differential diagnosis in patients who present inflammation and abscess formation after hernia repair. Besides, for patients with suspected intestinal fistula after hernia repair, laparoscopic exploration should be given priority, and the mesh removal approach should be tailored according to the results of laparoscopic exploration.

## Introduction

Inguinal hernia is the most common type of hernias in clinic practice. Since Bassini reported his hernioplasty in 1884, great progress has been made. In 1958, Usher et al. successfully applied a “Marlex” mesh to hernioplasty (1). Later in 1986, Lichtenstein proposed the “the tension-free hernioplasty” project, which had reduced the recurrence rate of hernia from 10–15% to 1–2% (2–4). Therefore, the application of mesh has become the standard for hernia repair surgery. However, postoperative mesh-related infection bothers both surgeons and patients because it is complicated to manage. When conservative treatment fails, it is inevitable to remove the infected mesh (5). Traditionally, the infected mesh is removed through an open approach. However, open debridement is very difficult and complicated, especially when late-onset mesh infection or fistula formation due to mesh migration and erosion of adjacent organs (6). The limited literature reports on late-onset intestinal leakage after inguinal hernioplasty make it extremely difficult for surgeons to make treatment decisions. This report presents a case of ileocutaneous fistula 8 years after plug repair with polypropylene mesh. Furthermore, available literature has been reviewed to provide a reference for the clinical diagnosis and the treatment of late-onset small intestinal fistula after inguinal hernioplasty.

## Case Presentation

**Figure 1:**
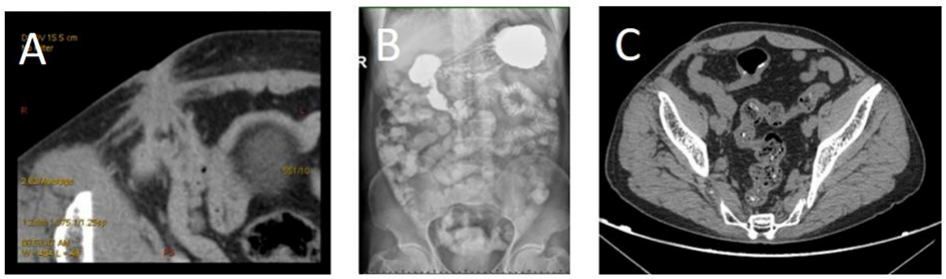
Images of representative abdominal CT and gastrointestinal radiography. **(A)** Infectious lesions in the right groin area, sinus formation, and involvement of the adjacent ileum; **(B)** no obvious signs of contrast agent leakage on barium meal imaging of the whole digestive tract; **(C)** 2 months after the surgery, the lesion in the right groin area is absorbed and narrowed, the sinus tract is unclear, and part of the intestinal canal in the abdominal cavity showed a metal anastomosis.

A 67-year-old male was sent to the Department of General Surgery for medical services due to skin ulceration with pus in the right groin area for 1 month. Psychical examination revealed a 1 × 2 cm ulceration in the right groin area, and the surrounding skin was red and swollen. Pus discharge from the fistula was observed when pressing the groin area, while the infected mesh was not observed. The patient had a plug repair with polypropylene mesh of right inguinal hernia 8 years ago. Gastrointestinal angiography showed no extravasation of contrast agents in the digestive tract (Figure 1). CT found skin sinus in the right groin area invaded the ileum (Figure 1). Therefore, based on the preoperative radiological images, the patient was diagnosed with mesh infections.

**Figure 2:**
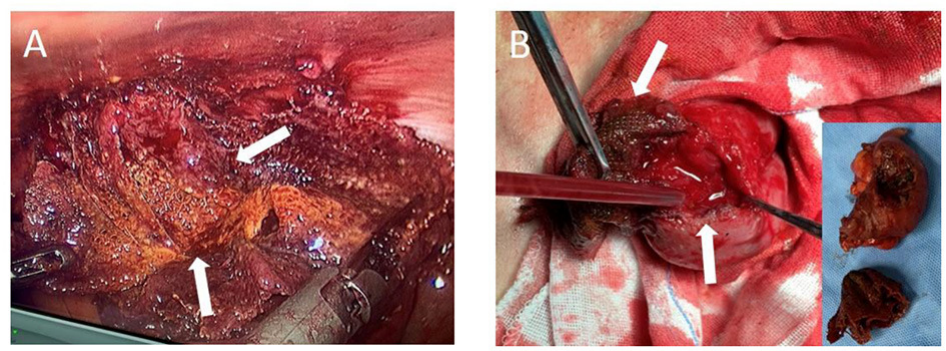
Intraoperative photographs. **(A)** Laparoscopic mesh and part of the ileum (white arrow), **(B)** Laparotomy mesh and part of the ileum (white arrow), and the embedded image shows the excised mesh and partial ileum specimen.

**Figure 3:**
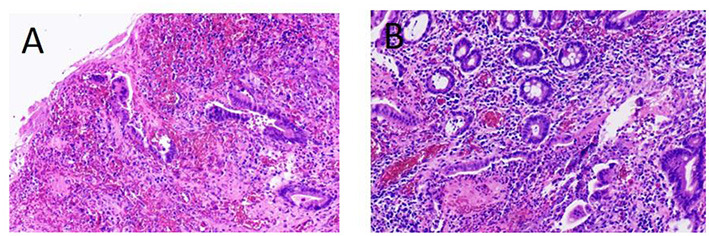
Postoperative pathology (HE staining) reveals intestinal vasodilation, congestion, edema, fibrous tissue hyperplasia, scattered infiltration of inflammatory cells, and partial mucosal defects accompanied by ulcer formation. **(A)** ×40 and **(B)** ×100 magnification, respectively.

Subsequently, the patient signed informed consent and took a laparoscopic exploration. Under laparoscopy, the ileum below the fistula closely adhered to the abdominal wall. After gently separating the bowel loop, a defect area of approximately 2 × 3 cm was observed on the surface of the ileum. The pus was aspirated immediately to avoid contamination of the abdominal cavity, and a sample was collected for culturing (Figure 2). According to the intraoperative evaluation, the male was diagnosed with enterocutaneous fistula. The surgery was converted to laparotomy, and the plug was found embedded in the ileum (Figure 2) and completely removed. Both ends of the ileum gap were cut with a linear cutting stapler to perform an ileum side-to-side anastomosis. The specimens were examined for pathology analysis, and the peritoneal flap was closed with 3–0 absorbable consecutive sutures. Postoperative pathology showed changes in intestinal inflammation, partial mucosal defects accompanied by ulcer formation (Figure 3). The patient was discharged 2 weeks after surgery with no postoperative complications. Follow-up at the sixth month revealed that the wound was completely healed and there was no sign of hernia recurrence.

## Discussion

Due to the significantly reduced recurrence rates, the appliance of mesh has become the standard for inguinal hernioplasty. Subsequently, increasing reports of complications caused by mesh infection, including wound infection, hematoma, chronic pain, and intestinal fistula, have been reported. Late-onset mesh infection is rare in clinical practice, while late-onset mesh infection with intestinal fistula is even rarer, and its diagnosis and treatment are the most complicated (7). Late-onset mesh infections are often characterized by inflammation, inguinal pain, swelling, erythema, induration, chronic post-herniorrhaphy neuralgia, and fever (8). If not appropriately treated, mesh infections will develop into an abscess and form a fistula. Studies have shown that 10.6% (5/47) of patients with late-onset mesh infections can develop sinus for a period of 9–45 months (9). There are two hypotheses about intestinal fistula caused by late-onset mesh infections: 1. Biomaterial mesh triggers human innate immunity (10); 2. Peritoneal defect caused by incomplete peritoneal closure or peritoneal damage caused by excessive mesh tension, the mesh migrates, and directly contacts the intestine (11, 12). A review of the literature on mesh migration has shown that symptoms of mesh infections appear 1–20 years after surgery (13). In this case, ileocutaneous fistula appeared in the eighth year after the inguinal hernia repair. Suppose the cause of the migration is related to surgery, such as direct contact between the mesh and the organ or excessive tension caused by the mesh. In that case, postoperative symptoms may appear very early. However, in the case of symptoms appearing 8 years after surgery, it seems that it is not only the cause of surgery. We believe that changes in the positional relationship between the mesh and the organs over time bring the mesh into close contact with the intestine, for example, the tapered shape and weight of the plug cause the mesh to move in space, and cause inflammation and local infection of the ileum, resulting in the formation of sinus tract and development of intestinal cutaneous fistula.

Intestinal fistula associated with late-onset mesh infections after the inguinal hernia is often diagnosed by ultrasound or CT, colonoscopy, and gastrointestinal angiography (14, 15). However, the scope and extent of infection and the relationship between the infected mesh and internal cavity organs are difficult to clearly visualize on preoperative imaging. As a minimally invasive and intuitive diagnostic method, laparoscopic exploration can diagnose and guide the treatment of intestinal fistula from the abdominal cavity without destroying the normal anatomical structure of the abdominal wall and adjacent organs (16). In our case, the patient underwent oral barium gastrointestinal angiography and CT scan before surgery. There was no obvious extravasation of contrast agents in gastrointestinal angiography. We considered it might be due to the sinus curvature that leads to sinus undeveloped. CT examination revealed the abnormal density in the right inguinal area, sinus formation, and ileum invasion, which provided great reference value for preoperative diagnosis. Intraoperative laparoscopic exploration revealed a defect area of approximately 2 × 3 cm on the surface of the ileum after gently separating the bowel loop, and part of the plug was embedded in the ileum lumen. Based on the intraoperative imaging examination and intraoperative exploration, the patient was diagnosed with the late-onset ileocutaneous fistula.

Currently, there is no consensus on the conservative treatment and debridement strategies for the secondary infection of the mesh (17, 18). Studies have suggested that when symptoms of mesh infection are found, it is recommended to take timely conservative treatment, including intravenous broad-spectrum antibiotics, wound care, and placement of percutaneous drainage tubes if necessary (19). When conservative treatment is ineffective for chills, fever, and wounds that are difficult to heal, surgical strategies should be adopted in time. This report discusses the case of mesh infection with intestinal fistula in the eighth year after inguinal hernia repair. The surgical strategy should be preferred. However, the challenge for surgeons is to completely remove the infected tissue to reduce the risk of infection recurrence or to choose to retain part of the mesh to prevent the hernia from recurring. Some literature has pointed out that the patch is not to replace the abdominal wall but to help rebuild the abdominal wall by stimulating the activity of fibroblasts (20). Acute inflammation occurred on the third day after surgery and was completely replaced by fibroblasts 2 months after surgery. In this case, the patient underwent inguinal hernia repair 8 years ago, and the toughness of the abdominal wall was strengthened according to laparoscopic exploration. So we chose to completely remove the infected mesh to reduce the risk of re-infection after surgery. Subsequently, we performed a segmental resection of the ileum and an ileum side-to-side anastomosis.

## Conclusion

Late-onset ileocutaneous fistula is a rare but serious complication of mesh infection and erosion that needs to be added to the differential diagnosis of patients who present inflammation and abscess formation after hernia repair. The surgeon should master the complex inguinal fascia structure to ensure correct anatomy along the preperitoneal space and fully free the preperitoneal space so that the mesh will not generate tension on the peritoneum and surrounding organs to make the peritoneum completely closed. Besides, for patients with suspected intestinal fistula after hernia repair, laparoscopic exploration should be given priority, and the approach of mesh removal should be tailored according to the results of laparoscopic exploration.

## Data Availability Statement

The original contributions presented in the study are included in the article/supplementary material, further inquiries can be directed to the corresponding author/s.

## Ethics Statement

The studies involving human participants were reviewed and approved by the Ethics Committee of the First Affiliated Hospital of Gannan Medical University. The patients/participants provided their written informed consent to participate in this study. Written informed consent was obtained from the individual(s) for the publication of any potentially identifiable images or data included in this article.

## Author Contributions

JZ designed the study and drafted the report. ZH and XL prepared the figure, did the literature search, and interpreted data. BC critically reviewed the report. All authors were involved in patient management and approved the final manuscript.

## Funding

This case report was supported by the Science and Technology Research Project of Jiangxi Provincial Education Department (grant no. 180797).

## Conflict of Interest

The authors declare that the research was conducted in the absence of any commercial or financial relationships that could be construed as a potential conflict of interest.

## Publisher's Note

All claims expressed in this article are solely those of the authors and do not necessarily represent those of their affiliated organizations, or those of the publisher, the editors and the reviewers. Any product that may be evaluated in this article, or claim that may be made by its manufacturer, is not guaranteed or endorsed by the publisher.
